# The Knight and the King: two new species of giant bent-toed gecko (*Cyrtodactylus*, Gekkonidae, Squamata) from northern New Guinea, with comments on endemism in the North Papuan Mountains

**DOI:** 10.3897/zookeys.562.6052

**Published:** 2016-02-10

**Authors:** Paul M. Oliver, Stephen J. Richards, Mumpuni, Herbert Rösler

**Affiliations:** 1 Division of Evolution, Ecology & Genetics, Research School of Biology, The Australian National University, Canberra, ACT 0200, Australia; 2 Department of Zoology, University of Melbourne, Parkville, Victoria 3052, Australia, and Department of Sciences, Museum Victoria, GPO Box 666, Melbourne, Victoria, Australia; 3 South Australian Museum, Adelaide, South Australia 5000, Australia; 4 Herpetology Division, Museum Zoologicum Bogoriense, Research Center for Biology, Indonesian Institute of Sciences (LIPI), Indonesia; 5 Senckenberg Naturhistorische Sammlungen Dresden, Museum für Tierkunde, Dresden, Germany

**Keywords:** Arc accretion, Endemism, Indonesia, lizard, orogeny, Papua New Guinea, Papua Province, Sepik Basin

## Abstract

The diverse biota of New Guinea includes many nominally widespread species that actually comprise multiple deeply divergent lineages with more localised histories of evolution. Here we investigate the systematics of the very large geckos of the *Cyrtodactylus novaeguineae* complex using molecular and morphological data. These data reveal two widespread and divergent lineages that can be distinguished from each other, and from type material of *Cyrtodactylus novaeguineae*, by aspects of size, build, coloration and male scalation. On the basis of these differences we describe two new species. Both have wide distributions that overlap extensively in the foothill forests of the North Papuan Mountains, however one is seemingly restricted to hill and lower montane forests on the ranges themselves, while the other is more widespread throughout the surrounding lowlands. The taxon endemic to the North Papuan Mountains is related to an apparently lowland form currently known only from Waigeo and Batanta Island far to the west – hinting at a history on island arcs that accreted to form the North Papuan Mountains.

## Introduction

Integrated morphological and molecular investigations of the exceptionally diverse biota of New Guinea are confirming that many nominally widespread species comprise multiple deeply divergent lineages (Donnellan and Aplin 1989; Oliver et al. 2013; Georges et al. 2014). In turn, as estimates of lineage diversity and phylogenetic relationships improve, so too does our understanding of patterns of regional and elevational endemicity and turnover, and the processes that have shaped them – most notably the complex geological history and extreme topography of New Guinea (Unmack et al. 2013; Toussiant et al. 2014).

The Bent-toed geckos (*Cyrtodactylus*) are the most species-rich radiation of geckos in the world (Wood et al. 2012; Uetz 2015). *Cyrtodactylus* diversity is concentrated in Indochina, South-east Asia and the Greater Sunda islands, however the clade extends from India and Sri Lanka in the west, the Himalayas in the north, through south-east Asia to the Philippines, Lesser Sundas, New Guinea and into northern Australia (Wood et al. 2012). Within the south-eastern region of this distribution New Guinea and surrounding islands are a centre of diversity, with an endemic radiation of at least 25 species, a majority of which have only been recognised in the last decade (Kraus and Allison 2006; Kraus 2007, 2008; Rösler et al. 2007; Oliver et al. 2008, 2009, 2012; Oliver and Richards 2012). Recent work has also indicated that the main Papuan lineage has evolved in at least one novel direction – it includes several lineages that are significantly larger than other *Cyrtodactylus* (SVL > 160 mm) (Zweifel 1980; Kraus 2007, 2008; Oliver et al. 2008; Bauer 2013; Oliver et al. 2014).

*Cyrtodactylus novaeguineae* Schlegel (1837) is the largest of these giant *Cyrtodactylus*, with a maximum recorded snout-vent length in excess of 170 mm (Zweifel 1980; Bauer 2013). While the type locality of *Cyrtodactylus novaeguineae* is in the Triton Bay area (now in Papua Barat Province) on the southern edge of the ‘Bird’s Neck’ in western New Guinea (Schlegel 1837), specimens from a wide range of localities both north and south of New Guinea’s Central Cordillera are currently assigned to this species (Brongesma 1934; Zweifel 1980; Kraus 2008) – generally on the basis that they possess enlarged tubercles extending onto and often across the posterior region of the throat (Zweifel 1980; Rosler et al. 2007; Kraus 2008).

Here we present an analysis of genetic and morphological variation within geckos referred to *Cyrtodactylus novaeguineae* from across New Guinea (with a focus on the much better sampled eastern half of the island). These data reveal two genetically and morphologically distinct lineages in northern New Guinea that are not conspecific with this nominal taxon - and which we therefore describe as new taxa. We also review the biogeography of these geckos in the context of recent phylogenetic investigations into the role that orogeny and arc accretion has played in shaping the biota of northern New Guinea.

## Materials and methods

### Sampling

DNA sequence data was amplified from tissues subsampled from frozen or ethanol collections lodged at the Australian Biological Tissue Collection (ABTC) in the South Australian Museum, the Museum Zoologicum Bogoriense (MZB), and the Bernice P. Bishop Museum (BPBM) (Appendix 1). Comparative material was examined at the following institutions: American Museum of Natural History (AMNH) – New York, Australian Museum (AMS) – Sydney, Bernice P. Bishop Museum (BPBM) – Honolulu, Museum of Comparative Zoology (MCZ) – Harvard University, Cambridge, Museum Zoologicum Bogoriense (MZB) – Bogor, and South Australian Museum (SAMA) – Adelaide (Appendix 2).

### Genetics

Sequence data from the NADH dehydrogenase subunit 2 (ND2) for 13 nominal *Cyrtodactylus novaeguineae* were aligned with a subset of Papuan *Cyrtodactylus* sequence data published elsewhere, and chosen to include all potential close relatives (Oliver et al. 2012). GenBank accession numbers and associated specimen data for newly amplified material are given in Appendix 2. Laboratory protocols largely followed Sistrom et al. (2009). ND2 and partial flanking tRNAs were amplified using the primers M112F (5’- AAGCTTTCGGGGCCCATACC-3’) and M1123R (5’- GCTTAATTAAAGTGTYTGAGTTGC –3’) designed in the flanking Methionine and Alanine tRNAs.

Our final alignment included up to 987 bp of data and was aligned using the MUSCLE algorithm (Edgar 2004) in Geneious version 6.0.5 (Biomatters 2012), and subsequently checked by eye. Phylogenetic trees were estimated using standard maximum Likelihood (RAxML v7.2.8; Stamakakis 2006) analyses implemented on the CIPRES web portal version 3.1 for online phylogenetic analysis (www.phylo.org/portal2). Data were not partitioned by codon (first, second and third base positions) and analyses were run using the default settings for RAxML on the CIPRES portal - the GTRGAMMA model of sequence evolution and ceasing bootstrapping when MRE-bootstrapping criteria had been reached.

### Morphology

Measurements taken with digital calipers to the nearest 0.1 mm largely follow Kraus (2006): snout-vent length (SVL), tail length (from the posterior edge of the vent to the tip of the tail) (TL), total length of original portion of tail (OT), trunk length from posterior edge of axilla to anterior edge of groin with limbs held at right angles (TrK), maximum head width (HW), maximum head height (HH), head length from tip of snout to anterior margin of ear opening (HL), distance from posterior edge of naris to eye (EN) (used as a proxy for snout-length), transverse diameter of eye (EYE), internarial distance (IN), transverse diameter of ear (EAR), forearm length from base of palm to outer edge of elbow (FA), and crus length from base of heel to outer edge of knee (CS).

We counted left and right enlarged supralabials to both the midpoint of the eye and to the rictus, left and right infralabials to rictus, dorsal tubercle rows between the lateral folds (not including the lateral fold) at the midpoint of body, ventrals at midpoint of the body in transverse series between ventral folds, the number of narrow lamallae distal to the inflection of the digit (not including the claw sheath), the number of wide subdigital lamellae proximal to the inflection of the joint under the first and fourth digits of the left manus and pes, precloacal and femoral pores where present, and postcloacal tubercles. Finally, we also recorded the extent of large tubercles on the lower jaw: absent, extending to the infra-angular region only, or extending across the throat.

## Results

### Genetics

**Figure 1.**
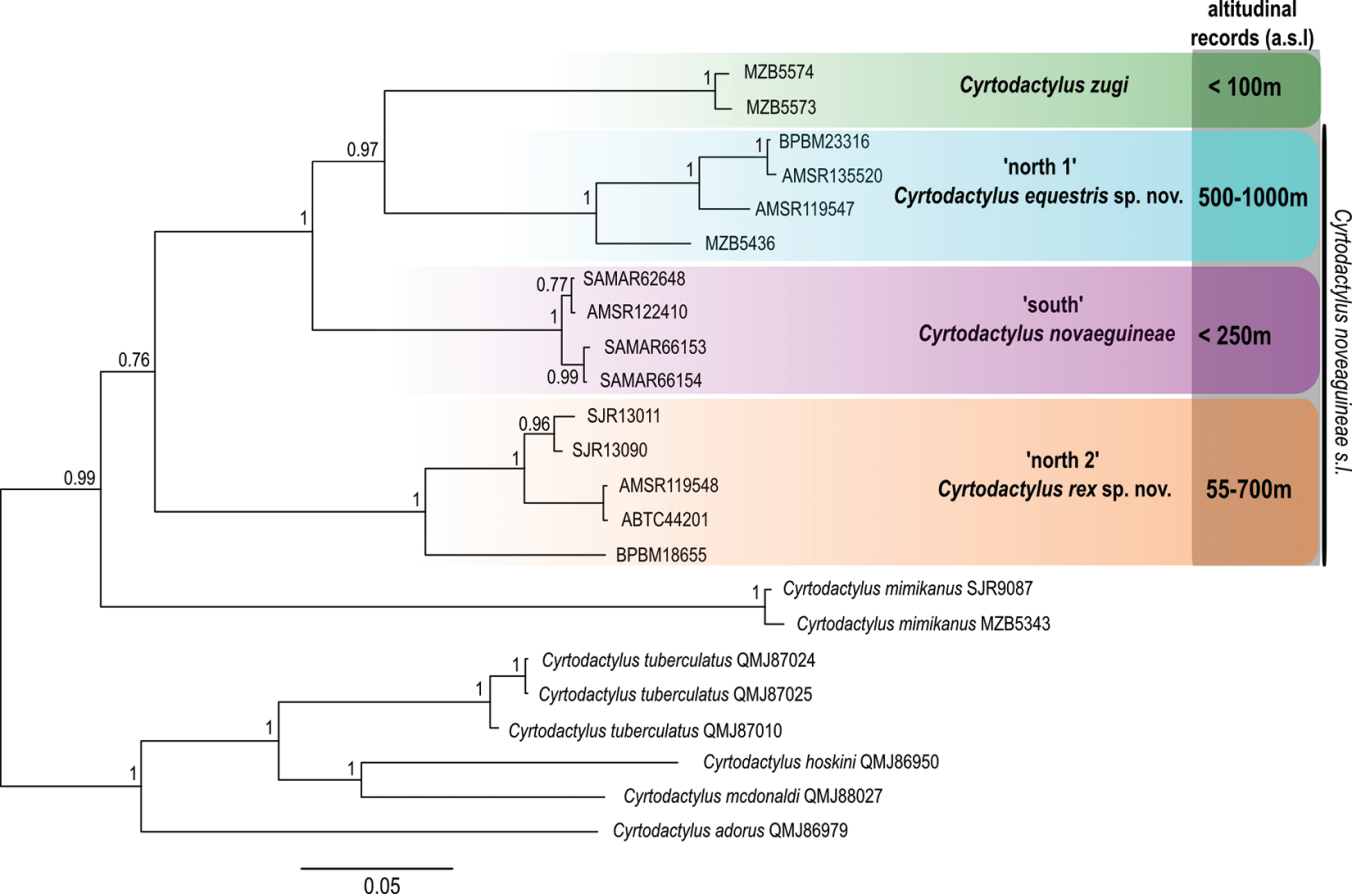
Maximum likelihood phylogeny for the *Cyrtodactylus novaeguineae* complex. The major lineages identified and the relationships between them estimated using RAxML and approximately 900bp of the mitochondrial *ND2* gene are shown. Posterior probability support values shown at key nodes. Known altitudinal distribution of all recognised species in metres above sea level (a.s.l.) also indicated.

We identify three major mitochondrial lineages of ‘*Cyrtodactylus novaeguineae*’: ‘south’ – from three sites to the south of the Central Cordillera in Western and Gulf Provinces of Papua New Guinea; ‘north 1’ – North Papuan Mountains (Foja, Bewani and Torricelli Mountains); and ‘north 2’ –northern lowlands and foothills of Papua New Guinea from close to the Indonesian border in Sandaun Province east as far as Morobe Province. A clade comprising ‘north 1’, ‘south’, and *Cyrtodactylus zugi* from Batanta Island off the western coast of New Guinea is strongly supported. Within this clade there is strong support for the close relationship of *Cyrtodactylus zugi* and ‘north 1’ (Figure 1). The ‘north 2’ lineage is more divergent, but a clade comprising all members of the *novaeguineae* group and its inferred closest relative *Cyrtodactylus mimikanus* (see Oliver et al. 2012) is supported. Mean levels of *ND2* sequence divergence between these four clades calculated using the Jukes Cantor Model ranges from (13.6–15.7%). There is evidence of additional mitochondrial structure in ‘north 1’ (mean 4.9%, max 7.5%) and ‘north 2’ (mean 4.7%, max 8.1%), but low diversity between samples of ‘south’ (mean 0.01%, max 0.01%).

### Morphology

**Figure 2.**
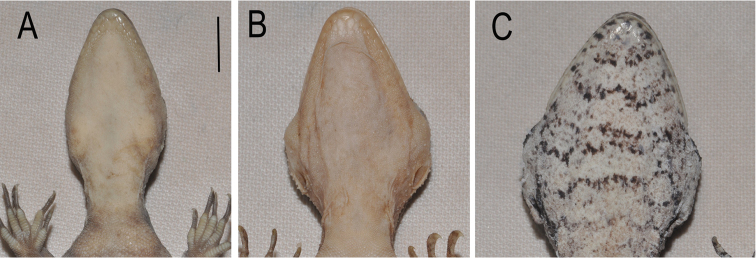
Throats of *Cyrtodactylus noveaguineae* and related species. **A** *Cyrtodactylus novaeguineae* (SJR10490/SAMA R66156) **B** *Cyrtodactylus equestris* sp. n. SJR6134/MZB lace 5436 (paratype), and **C** *Cyrtodactylus rex* sp. n. Scale bar = 1 cm. Note variation in relative width and colouration.

**Figure 3.**
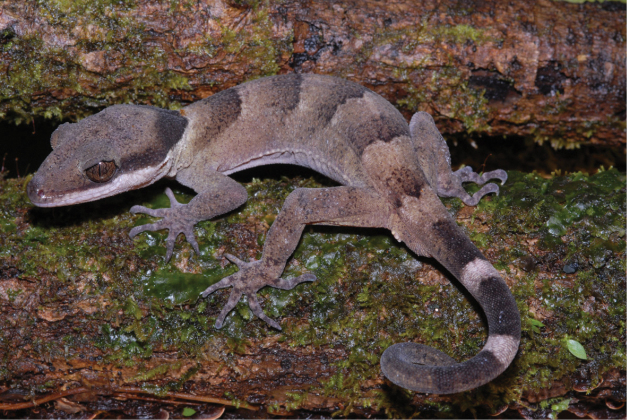
Genotyped *Cyrtodactylus novaeguineae* from southern slopes of the Central Cordillera of New Guinea. Photograph S. Richards.

**Figure 4.**
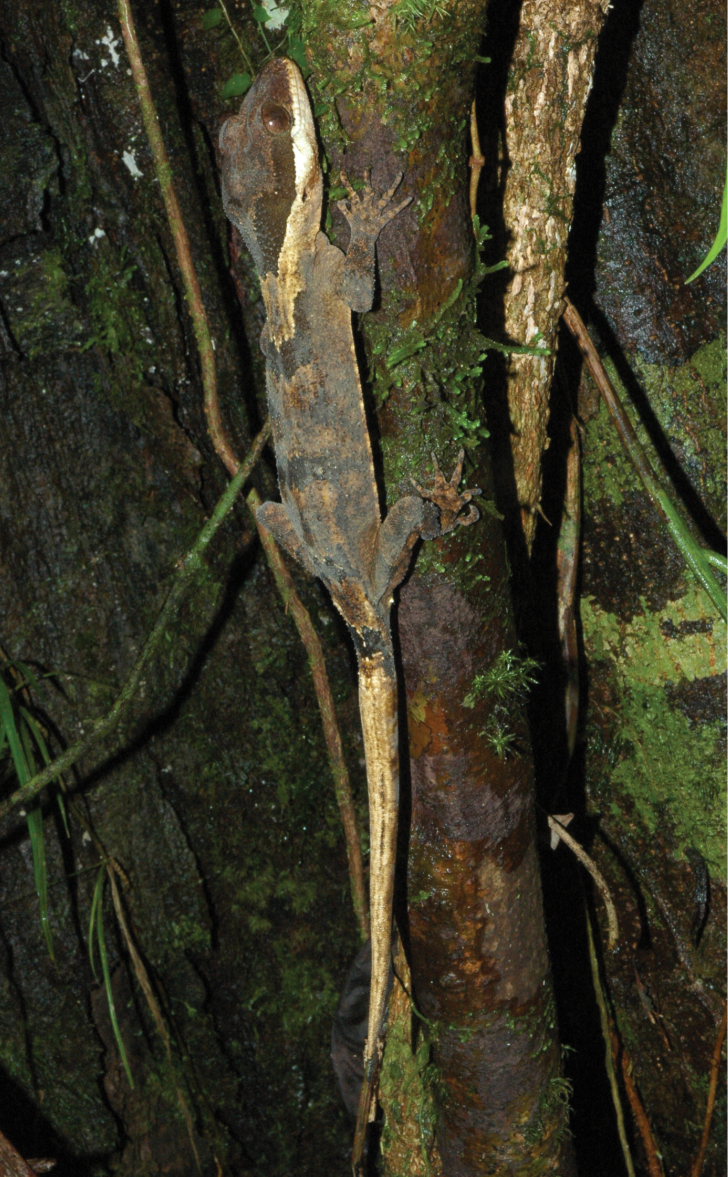
*Cyrtodactylus equestris* sp. n. in life. Paratype MZB lace 5435 from near Marina Valen Village, Papua Province, Indonesia. Photograph S. Richards.

**Figure 5.**
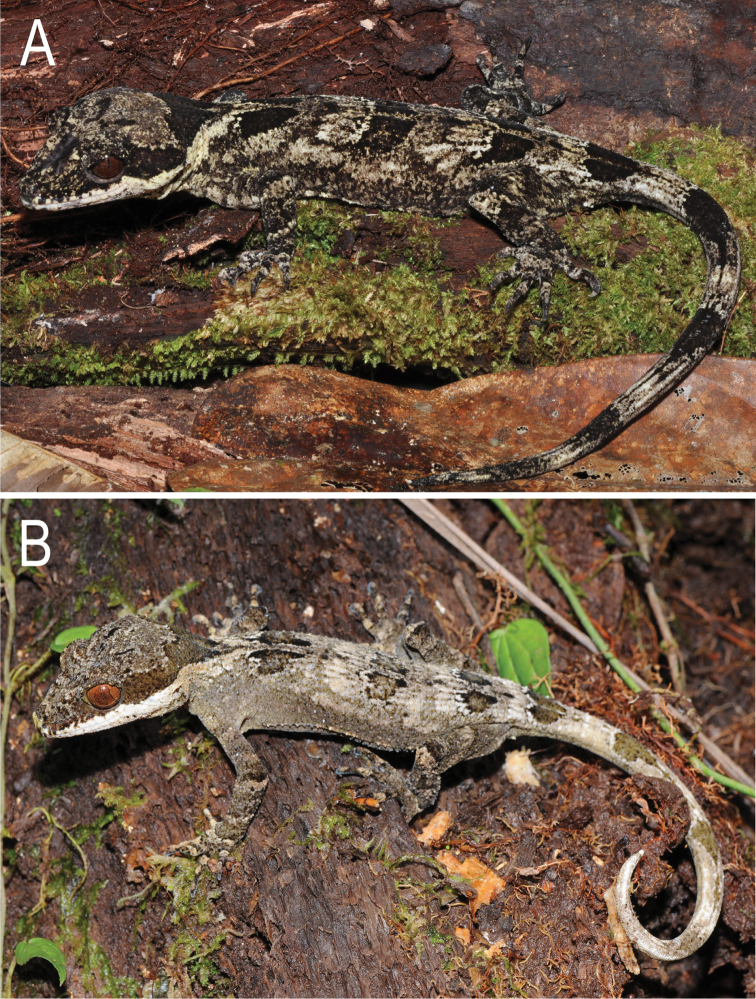
*Cyrtodactylus rex* sp. n. in life. **A** adult female holotype SAMA R67636 from the Sepik River Basin, East Sepik Province **B** juvenile paratype SAMA R67637 from Sepik River Basin, Sandaun Province. Photographs S. Richards.

Each of the three genetic lineages shows consistent differences in colour pattern, body size and aspects of scalation (see further details in Table 1, Figures 2–5, and comparisons below). The ‘south’ lineage is characterised by smaller size, narrow head (Figure 2), low number of ventral scales in transverse series, higher number of and darker and unbroken dorsal bands (Figure 3), plain venter and unbroken pore series in males. The ‘north 1’ lineage is of intermediate size, has a broad head (Figure 2), higher number of ventral scales, a dorsal colour pattern consisting of three relatively indistinct light brown transverse bands or patches on a light greyish brown background (Figure 4), a relatively plain venter with at most scattered small dark brown maculations, and a widely broken pore series in males. Finally, ‘north 2’ is distinctly larger, has a broad head (Figure 2), high number of ventral scales, a ‘messier’ three toned dorsal colour pattern comprising alternating but indistinctly defined regions of dark brown, medium grey and light grey to dirty off white (Figure 5), extensive amounts of dark-brown barring underneath the throat (Figure 2) and often also on the ventral surfaces of the body, and a generally continuous pore series in the males.

**Table 1.** Comparison of key morphological characters for members of the *Cyrtodactylus novaeguineae* complex. Key diagnostic traits for species are in bold.

Character	*Cyrtodactylus equestris* sp. n.	*Cyrtodactylus ‘novaeguineae*’	*Cyrtodactylus rex* sp. n.	*Cyrtodactylus zugi*
SVL (max)	**139 mm**	**129 mm**	**172 mm**	159 mm
HW/SVL	0.19–0.23	**0.17**–**0.19**	0.20–0.24	0.21-0.22
HH/HW	0.11–0.14	0.11–0.13	0.11–0.14	0.12-0.13
Dorsal tubercle rows	19–25	**21**–**22**	21–27	21-24
Ventrals	42–59	**31**–**44**	49–60	45-52
Supralabials	11–15	11–15	10–15	10–12
Infralabials	10–13	11–13	11–14	11–13
Extent of throat tubercles	usually across posterior throat	usually across posterior throat	usually across posterior throat	**none**
Number of pores	up to 39	up to 43	up to 38	unknown
Pore arrangement	**Tripartite**	continuous	continuous	unknown
Ventral pattern	sparse maculations	**unpatterned**	**dark brown reticulations**	very sparse maculations
Darkest band colour	**medium brown**	dark brown	dark brown	dark brown
Dark dorsal markings on body (including nuchal band)	3	**4–5**	4	4
Dark markings > .5× width of body	Some	**all**	some	some

## Systematics

Concordant patterns of genetic and morphological variation indicate that at least three evolutionarily distinct lineages (species) have been confounded within *Cyrtodactylus novaeguineae*. No genetic samples are available from the vicinity of the type locality so determining which, if any, of these populations represents true *Cyrtodactylus novaeguineae* relies on comparisons of morphology. The two male syntypes of *Cyrtodactylus novaeguineae* (RENA (formerly RMNH) 2708A–B) are of relatively small size (SVL 115 and 129 mm) with narrow heads (HW/SVL 0.18 and 0.19), and continuous to near-continuous pore series (divided only by one or two medial scales).This combination of morphological characters clearly distinguishes the types from both of the ‘north’ lineages, but does not distinguish them from the lineage we refer to here as ‘south’.

**Figure 6.**
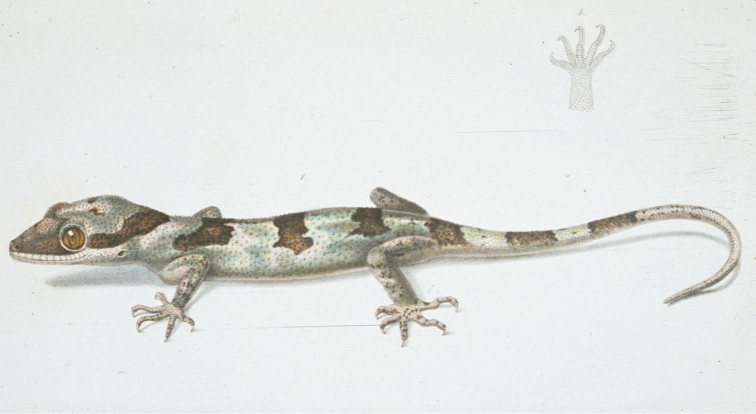
Reproduction of plate of *Cyrtodactylus novaeguineae* from the original description (plate published in 1844, several years after original description of 1837).

A colour plate accompanying the description of *Cyrtodactylus novaeguineae* (Schlegel, 1834) – presumably of one of the syntypes although this is not clear – shows three continuous and clearly defined brown dorsal bands (Figure 6). Recently collected specimens of ‘south’ also have strong dorsal bands, but usually have four instead of three. Unfortunately the colour patterns illustrated by Schlegel are no longer evident on the types and Schlegel does not report whether the non-illustrated material had a different number of dorsal bands. Nearly one thousand kilometres separates the type locality of *Cyrtodactylus novaeguineae* from the nearest locality for genetically typed ‘south’ lineage. Brongersma (1934) lists additional samples from southern New Guinea, especially from around the Lorentz River, however there are again few recent collections from this area and none with matching tissue samples. Given the limited morphological divergence between material from ‘south’ populations and the types of *Cyrtodactylus novaeguineae*, and the lack of genetic and colour pattern data for the population from the type locality, we conservatively consider the ‘south’ population to represent easternmost populations of *Cyrtodactylus novaeguineae* at this stage.

The populations from southern New Guinea that we here refer to *Cyrtodactylus novaeguineae* can be distinguished from other Papuan *Cyrtodactylus* by the following unique combination of characters – moderately large size (SVL to 129 mm), narrow head (HW/SVL 0.18–0.19), enlarged tubercles on the infra-angular region and often extending across the posterior region of the throat, mid dorsal tubercles in 21 to 22 rows at midpoint of body, subcaudal scales not transversely widened, moderate number of mid-body ventral scales (31–44) and a continuous or near-continuous, relatively straight, row of femoral and precloacal pores in adult males (up to at least 43 pores in total).

The two northern forms (‘north 1’ and ‘north 2’) differ from *Cyrtodactylus novaeguineae* from southern New Guinea (including the types) in having broader heads and in being of slightly to much larger size (Figure 2). The ‘north 1’ lineage also has a discontinuous series of femoral and preclocal pores divided by one or two regions of poreless scales. We consider that these characters, coupled with substantial genetic divergence between the northern lineages and southern *Cyrtodactylus novaeguineae*, and between ‘north 1’ and ‘north 2’ are sufficient to differentiate these two lineages and we present their formal descriptions below.

### Cyrtodactylus equestris sp. n.

Taxon classificationAnimaliaSquamataGekkonidae

http://zoobank.org/EF29B95D-5C28-4A26-B6BF-D9A566E79996

Figures 2 , 4

#### Holotype.

AMS R135520 adult male with everted left hemipenis and completely regrown tail, Papua New Guinea, Sandaun Province, Torricelli Mountains, Mt. Sumbau (3°23'S, 142°31'E, between 1000–1200 m a.s.l.), collected by P. German, 10 March 1990, with frozen tissue at the South Australian Museum (ABTC50282).

#### Paratypes

**(n = 6).** Papua New Guinea: AMS R119547 Sandaun Province, Torricelli Mtns, Wigote (3°25'S, 142°09'E), collected by T. Flannery, 20 July 1985; BPBM 23314–16 Sandaun Province, Torricelli Mountains, between 2.9–3.2 km east of Mt Sapau summit (3°23'27.0636"S, 142°31'47.028"E, 550–700 m a.s.l.), collected by F. Kraus between 23–25 May 2005. Indonesia: MZB lace 5435–6 Papua Province, Foja Mountains, camp above Marina Valen Village (02°22.230'S, 138°12.753'E; 500 m a.s.l.), collected by S. Richards and B. Tjaturadi between 17–22 July 2004.

#### Referred material

**(n = 5).** Papua New Guinea: AMNH 100050–1, Sandaun Province, Lumi (~530 m a.s.l.), collected by M. Lorenz; AMNH 100052 Sandaun Province, Mt Menawa, Bewani Mountains, collected by J. Diamond; AMNH 82360 Madang Province, Adelbert Mountains, Maratambu (~700 m a.s.l.), collected by E.T. Gillard; AMNH 103193 Madang Province, Adelbert Mountains, Wanuma (~700 m a.s.l.), collected by A.C. Zeigler. The last two specimens are listed as referred material because of taxonomic uncertainty (see below), while the remainder are relatively poor specimens.

#### Diagnosis.

A large *Cyrtodactylus* (SVL to 139 mm), with a moderately broad head (HW/SVL 0.19–0.22), enlarged tubercles on the infra-angular region and often extending across the posterior throat, mid-dorsal tubercles in 19 to 25 rows at midpoint of body, subcaudal scales not transversely widened, high number of mid-body ventral scale rows (42–59), femoral pores in two separated rows of 9–19, usually with a further medial precloacal row of 6–13 pores (up to 39 pores in total), venter relatively plain brown with at most scattered darker brown maculations, and dorsum with three distinct to indistinct medium-brown transverse bands on relatively plain light brownish-grey background.

#### Description of holotype.

A moderately large (113 mm SVL) and slender gecko. Head large (HL/SVL 0.28), moderately wide (HW/SVL 0.21) and clearly distinct from neck. Snout rounded in dorsal profile, broadly truncate in lateral profile, eye to naris distance longer than eye diameter (EN/EYE 1.4), loreal region slightly inflated, interorbital region and top of snout concave, canthus rostralis rounded, weakly defined. Eyes large (EYE/HL 0.26), pupil vertical, supraciliaries extending from anteroventral to posterodorsal edge of orbit, longest at the anterodorsal corner. Ear opening rounded, bordered by distinct dorsal skin fold.

Rostral rectangular, wider than high, with medial suture extending approximately halfway from dorsal edge towards ventral edge, bordered dorsally by two flattened nasals and single tiny internasal. Nares bordered by first supralabial (point contact), rostral, nasal, 2–3 enlarged postnasals and 2–3 tiny granular postnasals. Supralabials generally wider than high, 10 on right, 11 on left, 8 to midpoint of eye. Head, temporal and nuchal scales small and granular, interspersed with numerous enlarged weakly conical tubercles, approximately 3–4 times width of surrounding scales, on temporal and posterior nuchal regions. Enlarged infralabials slightly to much wider than high, 11 on right and 10 on left, bordered by rows of slightly enlarged scales that grade into small granular gular scales. Mental slightly wider than long, broadly triangular, but with distinctly concave edges at contact with postmentals, in contact with first infralabials. Scattered small conical tubercles (approximately twice size of surrounding scales) in the infra-angular regions of the lower jaw only.

Body moderately robust (TrK/SVL 0.44) with distinct ventrolateral folds. Moderately tuberculate, tubercles along lateral fold heterogeneous, up to 3 times larger than surrounding scales. Dorsum with approximately 23 rows (not including lateral fold) of often keeled tubercles up to 4 times width of surrounding granular scales. Ventral scales much larger than dorsal scales, increasing in size medially, arranged in approximately 39 rows at midpoint of body. Several continuous rows of enlarged femoral scales, posterior row extending almost to knee, distinctly larger and contrasting against granular posterior femorals. Precloacal pores in a series of 8, femoral pores in individual series of 15–16, respective series separated by 7 poreless scales.

Limbs moderately robust, forelimbs (FA/SVL 0.14) shorter and less robust than hindlimbs (CS/SVL 0.19). Lateral and dorsal surfaces of antebrachium and crus with numerous conical tubercles. Digits long and well developed, inflected at basal interphalangeal joints; subdigital lamellae smooth, rounded and expanded proximal to digital inflection (8-12-11-13-11 manus; 9-12-15-15-13 pes); narrow distal to digital inflection (9-10-11-11-11 manus; 7-12-12-14-13 pes) (counts not including ventral claw sheath); large recurved claws sheathed by a dorsal and ventral scale.

Tail almost completely regrown, scalation heterogeneous and irregular. Cloacal sacs swollen and prominent, each with 3 rounded cloacal spurs at anterior edge.

#### Measurements of holotype

**(in mm).** SVL 113, TL 97, OT 13, TrK 49.5, HW 23.4, HH 13.1, HL 32.2, EN 11.6, IN 4.2, EYE 8.3, EAR 2.0, FA 15.3, CS 21.5.

#### Color in ethanol.

Dorsal pattern consisting of alternating light brown and medium brown regions. Nuchal band medium brown, posterior edge triangular with thin continuous dark brown margin and extending along dorsum to level of forelimb insertion, anterior edge deeply notched and less clearly margined. Nuchal dark band bordered posteriorly by a deeply notched light brown band with distinct thin dark brown edging on medial anterior and posterior edges, and extending anteriorly onto lower jaw. Subsequent dark bands not deeply notched and less distinctly margined, but generally with at least some dark brown edging at their midpoint. Dorsal surface of head medium brown, darker anteriorly, without pattern, with the exception of a pair of small curved dark brown lines on the nape. Lower lateral region of head whitish brown, strongly demarcated against upper lateral and dorsal brown colouration. Ventral colouration dirty brown with scattered darker brown maculations on the throat and and across the venter. Limbs medium brown dorsally, slightly lighter ventrally, largely unpatterned except for scattered dark maculations and very small blotches on the hindlimbs. Stub of original tail medium brown dorsally with a pair of smeared very dark brown markings. Regrown tail plain light brown on all surfaces.

#### Variation.

The type series includes 4 adult males (with fully expressed pore series) varying from 113–129 mm SVL, two adult females both of 139 mm, and one juvenile male of 104 mm. Mensural data for the type series are summarized in Table 2. Supralabials to center of eye 8–10, to rictus of jaw 11–15, Infralabials 10–13. Fourth toe wide lamellae 11–13, fourth toe narrow lamellae 11–13, mid-belly scale rows 39–59, and maximum number of dorsal tubercle rows 19–23. Cloacal spurs 3–4, expressed precloacal pores from 6–8, femoral pore series from 8–15, total number of pores 24–35.

**Table 2.** Measurements for the type series of *Cyrtodactylus equestris* sp. n.

	AMS R135520	BPBM 23314	BPBM 23316	BPBM 23315	MZB lace 5436	MZB lace 5435	AMS R119547
Sex	m	m	M	m	f	f	juv
SVL	113	129	129	125	139	139	104
TL	97	148	104	151	119	129	135
OT	13	148	18	151	22	69	135
TrK	49.5	57.7	57.1	56.7	60.8	61.0	51.3
HW	23.4	26.5	26.8	24.4	26.8	29.7	20.6
HH	13.1	14.2	15.0	13.9	16.1	17.1	13.1
HL	32.2	34.1	35.3	32.7	34.8	39.1	27.6
EN	11.6	12.4	13.1	11.6	11.4	4.5	10.4
IN	4.2	5.2	5.1	4.7	4.8	5.1	3.7
EYE	8.3	7.7	7.9	8.1	7.8	8.7	7.7
EAR	2.0	1.8	1.1	1.7	2.1	1.7	1.7
FA	15.3	19.5	19.7	19.1	20.0	19.9	14.9
CS	21.5	22.1	23.1	22.2	23.0	25.5	18.7

Dorsum generally with alternating transverse regions of light and medium brown, however the width and distinctiveness of these region varies. Some variation in the intensity of colouration may be ontogenetic. On the largest specimens the medium brown regions are relatively narrow, and not or only weakly defined by dark brown edging, giving the overall impression of a somewhat faded pattern. On smaller specimens the transverse bands are more distinct and strongly defined. An indistinct trace of medium brown mottling or barring is also sometimes apparent on the dorsal and lateral surfaces of the hindlimbs. Venter medium to light brown, sometimes with very scattered darker brown maculations. Original tails with alternating medium-brown dorsal blotches and light-brown to creamish regions, border between colours often sharply defined by dark- brown edging. Regrown tails creamish or light brown with at most a few very indistinct light brownish streaks and patches. Iris in life deep chestnut brown with dark brown vermiculations (Figure 4).

#### Comparisons.

*Cyrtodactylus equestris* sp. n. can be distinguished from most other *Cyrtodactylus* by its large size (males to 129 mm, females to 139 mm), including all species from west of Lydekker’s Line (maximum size <130 mm). It can be differentiated from the other large Papuan taxa as follows. *Cyrtodactylus equestris* sp. n. differs from *Cyrtodactylus loriae* and *Cyrtodactylus serratus* in having enlarged tubercles on the infra-angular region and often extending across the throat (vs. absent), a lower number of pores (up to 39 vs. up to 81) in a discontinuous series (vs. continuous), and in lacking enlarged tubercles extending the length of the tail (vs. *Cyrtodactylus serratus* only). *Cyrtodactylus equestris* sp. n. differs from members of the *Cyrtodactylus lousiadensis* group (*Cyrtodactylus epiroticus*, *Cyrtodactylus klugei*, *Cyrtodactylus lousiadensis*, *Cyrtodactylus murua*, *Cyrtodactylus robustus*, *Cyrtodactylus salomonensis* and *Cyrtodactylus tripartitus*) in its smaller subcaudal scales, in having tubercles on the infra-angular region and throat, and in its more poorly defined light-brown bands or blotches on the dorsum (vs. strongly defined and unbroken transverse brown banding). *Cyrtodactylus equestris* sp. n. differs from *Cyrtodactylus zugi* in its smaller size (139 vs. 159 mm SVL), more extensive tuberculation that usually extends across the throat (vs. on infra-angular region only), and dorsal colour pattern on torso consisting of light-brown transverse bands on a plain greyish-brown background (vs. alternating dark brown blotches on a mottled dark-grey and off-white background). *Cyrtodactylus equestris* sp. n. differs from *Cyrtodactylus irianjayaensis* by its smaller size (139 vs. 163 mm SVL), the presence of enlarged tubercules usually extending across the throat (vs. infra-angular region only) and its higher number of femoral and precloacal pores (24–39 vs. 7–16). *Cyrtodactylus equestris* sp. n. differs from other populations of *Cyrtodactylus* here referred to *Cyrtodactylus novaeguineae* (both syntypes and genotyped material) in its wider head (HW/SVL 0.19–0.23 vs. 0.18–0.19), larger size (SVL 139.0 vs. 129.0) and tripartite femoral and precloacal pore arrangement (vs. continuous or at most one poreless intervening scales).

#### Distribution and natural history.

**Figure 7.**
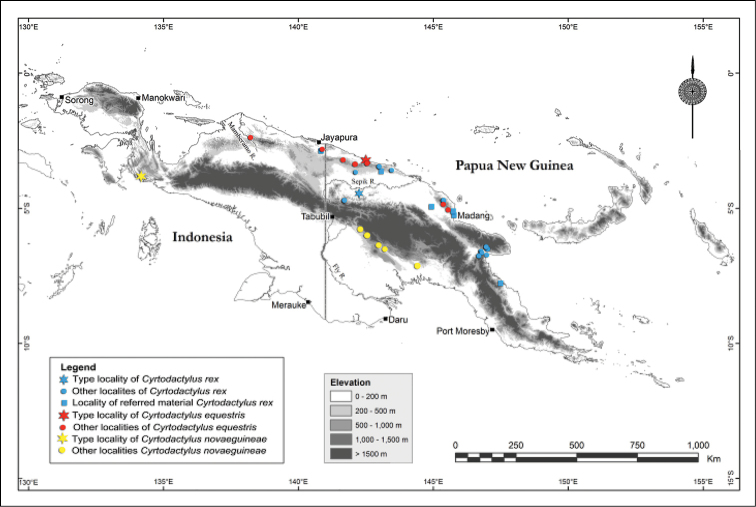
Distribution map for *Cyrtodactylus novaeguineae*, *Cyrtodactylus equestris* sp. n. and *Cyrtodactylus rex* sp. n.

Known from scattered localities in the Foja, Torricelli and possibly the Adelbert Ranges (see below) of northern New Guinea (Figure 7). Specimens for which detailed information is available were collected in relatively undisturbed hill or lower montane forest between 500–1200 m a.s.l.

#### Etymology.

*Equestris* latin for knight, in reference to the relative size of this species – large for the genus, but still subordinate to some of its near relatives.

#### Comments.

The referred material include two specimens in the American Museum of Natural History (AMNH 82360, AMNH 103193) from separate localities in the Adelbert Ranges, Morobe Province. These specimens have plain venters and two-toned brown and light brown dorsal colouration. On this basis they do not conform with ‘north 2’ (the only other member of the *Cyrtodactylus novaeguineae* complex from northern New Guinea) and are tentatively assigned to *Cyrtodactylus equestris* sp. n. However these localities are separated from the other localities in the North Papuan Mountains by the low swampy country around the Sepik River, and the single male from this region has a bipartite pore arrangement (vs. clearly tripartite). Fresh material and genetic samples are required to confirm the taxonomic status of these easternmost populations.

### Cyrtodactylus rex sp. n.

Taxon classificationAnimaliaSquamataGekkonidae

http://zoobank.org/10AED2AF-65E2-43FF-ACE8-A3A29AF6B9E7

Figures 2 , 5

#### Holotype.

SAMA R67636 (Field number SJR13190), Papua New Guinea, East Sepik Province, un-named camp in Sepik River basin, (4°24'14"S, 142°17'33"E, 55 m a.s.l.), adult female, collected by S. Richards, 1 March 2011, tissue stored in ethanol at the South Australian Museum ABTC114693.

#### Paratypes

**(n =19).** Papua New Guinea: SAMA R67637 (SJR13011) Sandaun Province, Sepik River Basin, un-named camp (4°43'39"S, 141°47'08"E, 425 m a.s.l.), collected by S. Richards on 20 February 2010; BPBM 11522 Morobe Province, Oomsis Forestry Camp (6°41'54.1278"S, 146°48'56.412"E, 400 m a.s.l), collected by A. Allison 3 March 1988; BPBM 18655 Morobe Province, 8.4 km W of Mt Shungol summit (6°47'40.56"S, 146°40'49.98"E, 420 m a.s.l.), collected by F. Kraus 23 October 2003; BPBM 34719 Madang Province, Samorek village (4°42'38.0412"S, 145°24'51.3714"E, 690 m a.s.l.), collected by F. Kraus 1 October 2009; BPBM 34747 East Sepik Province, Joromba River, 16.25 km W of Wewak (3°34.732'S, 143°30.020'E, 227 m a.s.l.), collected by F. Kraus 25 September 2009; AMS R13025 Morobe Province, Lae (6°44'S, 147°00'E), collected by E.L. Troughton 18 May 1945; AMS R31940 Morobe Province, Lae Botanic Gardens (6°44'S, 147°00'E), collected by E.L. Troughton 5 September 1969; AMS R129290 East Sepik Province, Maprik (3°25'S, 143°02'E), collected by W.H. Ewers 5 November 1964; AMS R119548–50 Sandaun Province, Torricelli Mts,Wigote (3°39'S, 142°09'E), collected by T. Flannery 22 July 1985; AMNH 92341–2 Morobe Province, Oomsis Creek (6°41'S, 146°48'E), collected by H.M. Van Deusen April 1959; AMNH 95165–8 Morobe Province, Lae (6°44'S, 147°00'E); AMNH 95169 Morobe Province, Busu River, 8 mi. N of Lae; AMNH 95170 Morobe Province, 13 mi. N of Lae, previous six specimens collected by R. Zweifel & G. Sluder July–August 1964.

#### Referred material

**(n = 7).** Papua New Guinea. AMNH 95171 East Sepik Province, Maprik (3°38'S, 143°03'E); AMNH 100048–9 Sandaun Province, Lumi (3°28'S, 142°02'E); AMNH 104871 Madang Province, Alexishafen (5°05'S, 145°48'E); AMNH 103194 Madang Province, Kaibugu (4°54'S, 144°57'E); MCZ 142462 Madang Province, 4 mi. S Madang; MCZ 96201 Morobe Province, Morobe town (7°45'S, 147°36'E).

#### Diagnosis.

A very large *Cyrtodactylus* (SVL to 172 mm), with a very broad head (HW/SVL 0.20–0.24), enlarged tubercles across the infra-angular region and often extending across the throat, mid-dorsal tubercles in 21 to 27 rows at midpoint of body, subcaudal scales not transversely widened, high number of mid-body ventral scales in transverse series (49–60), moderate number of femoral and precloacal pores (20–38) in a nearly continuous chevron, narrow dark brown barring on the throat, labials and often venter, and dorsal colour pattern on torso including indistinctly defined alternating dark-brown, medium-brown and whitish regions.

#### Description of holotype.

A very large (169 mm SVL) and robust gecko. Head very large (HL/SVL 0.27), very wide (HW/SVL 0.23) and clearly distinct from neck. Snout longer than eye diameter, eye to naris distance longer the eye (EN/EYE 1.4), curved in dorsal profile, broadly truncate in lateral profile, mid-loreal region slightly inflated, interorbital region and top of snout slightly concave, canthus rostralis weakly defined. Oval patch of skin missing from top of snout. Eyes large (EYE/HL 0.24), pupil vertical, supraciliaries extending from anteroventral to posterodorsal edge of orbit, longest at the anterodorsal edge. Ear opening roughly circular, bordered by distinct dorsal skin fold.

Rostral broadly rectangular, approximately 1.5 times wider than high with medial suture extending approximately 60% from dorsal edge towards ventral edge, bordered dorsally by two nasals and three smaller internasals. Nares bordered by first supralabial (point contact), rostral, nasal, and series of five to eight granular postnasals. Supralabials generally slightly wider than high, 13 right, 14 left, 10 to midpoint of eye. Head, temporal and nuchal scales small and granular with conical tubercles approximately 2–3 times width of surrounding scales densely distributed across the temporal and nuchal regions. Enlarged infralabials to rictus 14 right, 13 left, anterior infralabials higher than wide, posterior infralabials wider than high, infralabials bordered by rows of enlarged scales that grade into small granular gular scales. Mental triangular, approximately as wide as long, bordered by first infralabials and two pentagonal postmentals. Numerous wide flat tubercles present across posterior region of throat.

Body robust (TrK/SVL 0.43) with distinct ventrolateral folds. Skin heavily tuberculate dorsally and laterally, 33–34 prominent enlarged tubercles along lateral folds, dorsum with up to 23 rows (not including lateral fold) of enlarged conical tubercles up to four times width of surrounding small and granular scales. Ventral scales larger than dorsal scales, increasing in size medially, arranged in approximately 54 rows at midpoint of body, one or two poorly defined rows of enlarged ventral tubercles present just inferior to the lateral fold. Enlarged precloacal and femoral scales in three rows, posterior row longest (47 scales) and extending laterally approximately two thirds length of femur, medial scales distinctly larger.

Limbs robust, forelimbs shorter (FA/SVL 0.15) and less robust than hindlimbs (CS/SVL 0.17). Lateral and dorsal surfaces of hindlimbs with numerous enlarged conical tubercles. Digits long and well developed, inflected at basal interphalangeal joints; subdigital lamellae smooth, rounded and expanded proximal to joint inflection (11–12–13–15–11 manus; 10–14–14–15–9 pes); narrow distal to digital inflection (7–9–10–10–11 manus; 8–8–12–11–11 pes) (not including ventral claw sheath); large recurved claws sheathed by a dorsal and ventral scale.

Tail original, partially fractured approximately halfway from base, long and moderately robust, numerous low conical tubercles on dorsal and lateral surfaces close to base, but not extending beyond anterior third of tail, subcaudal scales enlarged, not wider than long, arranged in series 2–4 scales wide, 4 rounded cloacal spurs.

#### Measurements of holotype

**(in mm).** SVL 169, TL 177, OT 177, TrK 72.7, HW 38.1, HD HH 24.3, HL 45.1, EN 14.7, IN 6.4, EYE 10.6, EAR 3.2, FA 26.1, CS 29.3.

#### Color in ethanol.

Dorsum consists of alternating regions of dark greyish-brown, medium grey, and light-grey to dirty off-white. Four dark-brown regions most clearly defined, and consisting of three paired sets of oval, pentagonal and triangular blotches between fore- and hindlimbs, and an additional distinct dark-brown triangular nuchal patch anterior to insertion of forelimbs, and extending anterio-laterally as a stripe through eye and along dorsal edge of supralabials. Ventro-lateral regions of head with wide off-white stripe extending to lower edge of supralabials. Supraciliaries and dorsal tip of snout dark brown. Limbs and toes dirty grey with broad indistinct dark-brown bands on upper and lateral surfaces. Ventral ground colouration off-white with brownish tinge and extensive dark-brown flecks, often covering just a single scale, but also coalescing to form four distinct sets of jagged transverse bars on throat, and less prominent bars and ocelli on torso. Dorsal and lateral surfaces of tail dirty grey with four indistinctly edged dark-brown blotches or bands, and extensive smaller dark brown maculations, stripes or blotches. Subcaudal surfaces dark-brown with scattered lighter grey spots.

#### Variation.

The type series of 20 specimens includes five adult males with expressed pores (SVL 152–165 mm), 10 females (128–172 mm), and five juveniles or subadults (72–127 mm). Mensural data for the type series are summarized in Table 3. Supralabials counted to center of eye vary from 8–12, to angle of jaw 10–15, infralabials vary from 11–14, fourth toe wide lamellae 10–19, fourth toe narrow lamellae 8–16, mid-belly scale rows 39–60, number of rows of dorsal tubercles 20–27, cloacal spurs 2–6, and expressed femoral and precloacal pores in continuous or near continuous series of 28–38 (males only).

**Table 3.** Measurements for the type series of *Cyrtodactylus rex* sp. n.

	Males (n = 5)		Females (n = 10)		Immatures (n = 5)	
	Range	Mean	Range	Mean	Range	Mean
SVL	127–165	153	128–172	154.3	73–111	89.3
TL	16–162	118.0	23–177	122.9	74–112	94.0
OT	11–137	40.6	11–142	37.2	74–112	94.0
TrK	54.9–78.2	70.0	47.0-87.2	69.8	34.4–53.7	42.4
HW	27.1–35.0	32.5	28.6–38.1	33.4	15.1–23.1	18.9
HH	16.6–21.8	19.8	15.5–24.3	19.7	9.3–14.0	11.7
HL	36.5–43.3	41.1	35.6–45.6	41.4	20.6–29.9	25.2
EN	12.2–14.5	13.6	12.1–15.1	13.8	7.1–10.7	8.7
IN	4.9–6.4	5.8	5.2–6.4	5.9	2.8–4.4	3.7
EYE	8.7–10.4	9.5	8.1–10.9	9.9	4.8–7.4	6.4
EAR	1.5–3.9	2.4	1.8–4.2	2.6	1.0–1.7	1.5
FA	20.5–24.2	23.0	18.6–26.1	22.3	9.9–16.4	12.8
CS	23.6–29.5	27.2	22.8–31.0	27.0	11.8–18.7	15.0

Dorsal pattern always consists of indistinctly defined alternating regions of dark grey brown, medium brown and dirty off-white. Dark grey-brown markings usually most clearly defined, but showing extensive variation in shape and size - usually less than half width of torso, but occasionally wider and varying in shape from small diamonds, transverse bands to paired blotches or triangles. Dark brown ventral barring always present, but on some specimens restricted to throat only, while in others forming a network across throat and venter. All specimens with at least some indistinct dark brown barring on toes and four or five dark brown longitudinal blotches or bands on original tails. Iris in life brick red with extensive fine brown vermiculations (Figure 5).

All specimens heavily tuberculate with usually several indistinct rows of large tubercles extending as much as 10 mm inferior to lateral fold at midpoint of torso. Throat tuberculation varies in extent from a broad band spanning the posterior throat to concentrated in infra-angular regions and largely absent from the throat.

#### Comparisons.

*Cyrtodactylus rex* sp. n. is readily distinguished from most other *Cyrtodactylus* by its very large size (SVL up to 172 mm vs generally < 130mm). It further differs from the relatively small number of other large Papuan species as follows. *Cyrtodactylus rex* sp. n. differs from *Cyrtodactylus loriae* and *Cyrtodactylus serratus* in having enlarged tubercles on the infra-angular region and often extending onto and across the throat (vs absent from both regions), a lower number of pores (up to 38 vs. up to 81), and in lacking enlarged tubercles extending the length of the tail (vs. *Cyrtodactylus serratus* only). *Cyrtodactylus rex* sp. n. differs from members of the *Cyrtodactylus lousiadensis* group (*Cyrtodactylus epiroticus* (with which it is sympatric in Morobe Province), *Cyrtodactylus klugei*, *Cyrtodactylus lousiadensis*, *Cyrtodactylus murua*, *Cyrtodactylus robustus*, *Cyrtodactylus salomonensis* and *Cyrtodactylus tripartitus*) by its much smaller subcaudal scales, the presences of extensive tubercles on infra-angular region and often the throat, and in its much more poorly defined dark bands or paired blotches on the dorsum (vs. distinctly edged, unbroken transverse light and dark-brown bands). *Cyrtodactylus rex* sp. n. differs from *Cyrtodactylus zugi* by the presence of dark-brown barring on the throat and venter, tuberculation often extending across the throat (vs. on infra-angular region only), and dorsal colour pattern on torso consisting of alternating indistinct dark-brown, medium brown and whitish regions (vs. alternating dark-brown and off-white). *Cyrtodactylus rex* sp. n. differs from *Cyrtodactylus irianjayaensis* by the presence of dark-brown barring on the throat and venter (vs. plain and unpatterned), tubercles often extending across the throat (vs. infra-angular region only), dorsal colour pattern on torso consisting of alternating indistinct dark-brown, medium brown and whitish regions (vs. very wide brown transverse blotches on a lighter greyish brown ground colour), and higher number of femoral and precloacal pores (21–38 vs. 7–16). *Cyrtodactylus rex* sp. n. differs from all populations referred to *Cyrtodactylus novaeguineae* in its wider head (HW/SVL 0.21–0.24 vs. 0.18–0.19) and larger size (SVL 172 vs. 129 mm), and differs from *Cyrtodactylus equestris* sp. n. in its larger size (SVL 172 vs. 139 mm), in having a continuous (or nearly so) row of femoral and precloacal pores, presence of dark barring on the throat and ventral surfaces of body (vs. absent), and ‘messier’ dorsal colouration of alternating indistinct dark-brown, medium brown and whitish regions (vs. light-brown transverse bands or blotches on relatively plain light brownish-grey background).

#### Distribution and natural history.

Widespread in northern Papua New Guinea, extending from Sandaun Province in the west to Morobe Province in the east (Figure 7). Photographs of a specimen from the vicinity of Senggi Village to the south of Jayapura in West Papua Province (kindly provided by Burhan Tjaturadi), indicate that this species also occurs in adjacent parts of Indonesian New Guinea.

The holotype was collected on a low ridge in Sago-dominated swamp forest. Other specimens were collected in lowland and foothill forest at altitudes ranging from near sea level up to 690 m a.s.l.

#### Etymology.

From the latin for king as it is the largest of the over 200 species of *Cyrtodactylus*, and amongst the largest of all known geckos (Bauer 2013).

## Discussion

The complex geological history of New Guinea has played a major role in shaping Papuan biodiversity (Toussaint et al. 2014). The high Central Cordillera is the dominant mountain complex in New Guinea, however the lower and smaller upland regions of the North Papuan Mountains also have an endemic biota that remains poorly understood (Helgen 2007; Richards et al. 2009; Kraus and Myers 2012). The North Papuan Mountains are derived from the (ongoing) accretion of formerly isolated island arcs onto the northern edge of New Guinea (Polhemus 2007). One key question is the extent to which endemism in these ranges is attributable to dispersal from other (relatively distant) montane habitats, persistance of a previously insular biota of terranes, or localised in situ transitions from surrounding lowland habitats (Toussaint et al. 2014).

The two new geckos described here have overlapping, yet somewhat complementary distributions: *Cyrtodactylus equestris* sp. n. is seemingly restricted to hill and lower montane forests on the North Papuan Mountains themselves, while *Cyrtodactylus rex* sp. n. is more widespread throughout the surrounding lowlands (Figure 7). *Cyrtodactylus equestris* sp. n. is the second *Cyrtodactylus* that has only been recorded from hill and low montane forest on the North Papuan Mountains – the other being *Cyrtodactylus boreoclivus* from the Foja, Torricelli and Bewani Mountains (Oliver et al. 2011). Sampled populations of both of these apparent North Papuan Mountain endemics show levels of mitochondrial genetic diversity (< 10%) consistent with the hypothesis that they represent single (ableit structured) species and suggest some degree of historical connectivity between the ranges that comprise the North Papuan Mountains (Oliver et al. 2012, this study). The as yet ungenotyped populations referred to *Cyrtodactylus equestris* sp. n. from the Adelbert Mountains in the east may prove to be an exception to this.

In contrast to their broadly overlapping intraspecific distribution, the distribution of sister lineages to these two North Papuan Mountain *Cyrtodactylus* differs. On the one hand *Cyrtodactylus boreoclivus* is closely allied to *Cyrtodactylus medioclivus*, an allopatric lower montane form currently known only from a small area of the Central Cordillera (Oliver et al. 2012). On the other hand, records of the inferred relatives of the newly described *Cyrtodactylus equestris* sp. n. (*Cyrtodactylus novaeguineae*, *Cyrtodactylus rex* sp. n. and *Cyrtodactylus zugi*) are concentrated in lowland habitats spanning New Guinea, and suggest that the upland habitat association of this taxon is derived. The putative sister taxon *Cyrtodactylus zugi* is currently known only from other still discrete northern terranes – the islands of Batanta and probably Waigeo (Oliver et al. 2008). Batanta and especially Waigeo have moved significantly westward through the Miocene and Pliocene (Polhemus 2007) – highlighting the possibilty that the evolutionary history of the clade comprising *Cyrtodactylus equestris* sp. n. and *Cyrtodactylus zugi* may be linked to the northern arc islands.

The contrasting distribution of sister lineages in the North Papuan Ranges suggests endemism is accumulating through multiple processes - colonisation by taxa already associated with hill and lower montane habitats from the older Central Cordillera (eccentric endemism), accretion of pre-existing island arc biotas, and potentially even de novo shifts up elevational gradients within otherwise lowland lineages (centric endemism) (Merckx et al. 2015) and complements recent work addressing the same question in water beetle (Toussaint et al. 2014).

*Cyrtodactylus* is also an exceptionally species rich genus of lizards with over 200 recognised species (Uetz 2015), however, how potentially key factors such as competition, ecological diversification, isolation and dispersal, have shaped the evolution of this diversity remain largely untested. Within New Guinea a number of lineages have larger body sizes than elsewhere, with the giant *Cyrtodactylus rex* sp. n. sitting at the apex of this trend. It has been suggested that this is indicative of a unique trajectory of ecological evolution in the Papuan region – potentially linked to either competitive and/or predatory release (Oliver et al. 2014). The ongoing elucidation of a suite of apparently lower montane and hill forest species suggests that altitudinal segregation has also played some role in mediating the accumulation of regional diversity in this lineage (Oliver et al. 2013; this paper), at least within the exceptionally complex orogeny of New Guinea, and potentially also other topographically variable tropical regions (Grismer et al. 2012).

## Supplementary Material

XML Treatment for Cyrtodactylus equestris

XML Treatment for Cyrtodactylus rex

## Appendix 1

**Registration numbers, GenBank numbers and localities for material included in genetic analyses.**

Ingroup. *Cyrtodactylus equestris*: BPBM 23316, JX440547, Papua New Guinea, 2.9–3.2km E of Mt Sapau summit; AMS R135520, KT835458, Papua New Guinea, Sandaun Province, Torricelli Mountains, Mt. Sumbau; AMS R119547, KT835457, Papua New Guinea, Sandaun Province, Torricelli Mtns, Wigote; MZB lace 5436, KT835459, Indonesia, Papua Province, Foja Mountains, Marina Valen Village; *Cyrtodactylus novaeguineae*: SAMA R62648, JQ820302, Papua New Guinea, Southern Highlands Province, Libano; AMS R122410, JQ820301, Papua New Guinea, Southern Highlands Province, Waro; SAMA R66153, JQ820304, Papua New Guinea, Western Province, upper Strickland basin; SAMA R66154, JQ820303, Papua New Guinea, Western Province, upper Strickland basin; *Cyrtodactylus rex*: SAMA R67637, KT835460, Papua New Guinea, Sandaun Province, Sepik River basin; SAMA R67636, KT835461, Papua New Guinea, Sandaun Province, Sepik River basin; AMS R119548, KT835462, Papua New Guinea, Sandaun Province, Torricelli Mts, Wigote; ABTC 44201 (no voucher), KT835463, Papua New Guinea, Sandaun Province, Torricelli Mts, Wigote; BPBM 18655, KT835463, Papua New Guinea, Morobe Province, 8.4km W of Mt Shungol summit.

Outgroups. *Cyrtodactylus adorus*: QM J86979, HQ401166, Australia, Queensland, Pascoe River; *Cyrtodactylus arcanus*: AMS R124559, JQ820314, Papua New Guinea, Madang Province, Bundi; *Cyrtodactylus arcanus*: (paratype) AMS R124560, JQ820319, Papua New Guinea, Madang Province, Bundi; *Cyrtodactylus epiroticus*: BPBM 18653, HQ401195, Papua New Guinea, Milne Bay Province, Mt Shungol; *Cyrtodactylus hoskini*: QM J86950, HQ401119 Australia, Queensland, Iron Range National Park; *Cyrtodactylus mcdonaldi*: QM J88027, HQ401150, Australia, Queensland, Parrot Creek Falls; *Cyrtodactylus mimikanus*: MZB lace 5343, JQ820316, Indonesia, Papua Province, Foya Mtns; SJR9807, KT835456, Indonesia, Papua Province, Foya Mtns; *Cyrtodactylus tuberculatus*: QM J87024, HQ401086, Australia, Queensland, Cooktown, Finch Bay; QM J87025, HQ401087, Australia, Queensland, Cooktown, Finch Bay; QM J87010, HQ401090, Australia, Queensland, Cooktown, Jenson’s Crossing; *Cyrtodactylus zugi*: MZB lace 5575, JQ820306, Indonesia, Papua Barat Province, Raja Ampat Archipelago, Batanta Island; MZB lace 5573, JQ820305 Indonesia, Papua Barat Province, Raja Ampat Archipelago, Batanta Island.

## Appendix 2

**Material examined for morphological comparisons.**

Institutional abbreviations are as follows: Australian Museum (AMS), American Museum of Natural History (AMNH), British Museum of Natural History (BMNH), Museum für Naturkunde, Berlin (ZMB), Museum of Comparative Biology, Harvard (MCZ), Museum Zoologicum Bogorienses (MZB), Naturalis, Amsterdam (RMNH), South Australian Museum (SAMA) and Western Australian Museum (WAM).

*Cyrtodactylus aaroni*: ZMB 62275 (holotype), ZMB 62273–4, ZMB 622737, ZMB 62756–7 (paratypes) Indonesia, Papua Barat Province, Wondiwoi Mountains; *Cyrtodactylus arcanus*: AMS R124599 (holotype), AMS R124599 (paratype) Papua New Guinea, Madang Province, Bundi; *Cyrtodactylus boreoclivus*: AMS R135519 (holotype) Papua New Guinea, Sandaun Province, Torricelli Mountains, Kukumbau area of Mount Sapau; MZB lace 7474–7475, SJR13593–13613 (field numbers, currently at MZB, to be lodged at South Australian Museum) Indonesia, Papua Province, Foja Mountains; *Cyrtodactylus capreoloides*: SAMA R62634 (holotype) Papua New Guinea, Southern Highlands Province, Moro; AMS R122412–122414 Papua New Guinea, Southern Highlands Province, Namosado; *Cyrtodactylus darmadvillei*: MZB lace 2005 Indonesia, Komodo National Park, Loh Liang; MZB lace 5297 East Flores, Mt Egon; *Cyrtodactylus derongo*: AMNH 103910 (paratype) Papua New Guinea, Western Province, Derongo Area, Alice River system; *Cyrtodactylus deveti*: MZB lace 6036 Indonesia, North Maluku Province, west Halmahera, Weda Village; MZB lace 6037 Indonesia, North Maluku Province, Halmahera, Tosoa Village; *Cyrtodactylus halmahericus*: MZB lace 6086 Indonesia, North Maluku Province, southern Ternate Island, Gambesi Village; MZB lace 6088, 6091–6092 Indonesia, North Maluku Province, Ternate Island, Faramadiahi Village; MZB lace 6095 Indonesia, North Maluku Province, east Halmahera, 17km from Lolobato National Park; MZB lace 1899 Indonesia, Maluku Province, Seram, Manusela National Park, Sasarata; MZB lace 2360 Seram, Kanike; MZB lace 2359, 2361 Seram, Solea; MZB lace 2362, 2363 Seram, Saunulu, Waikawa. *Cyrtodactylus irianjayensis*: MZB lace 5765 Indonesia, Papua Barat Province, Salawati Island. Cyrtodactylus cf. irianjayensis: MZB lace 2297 (seven specimens), 2298 (five specimens) Indonesia, Papua Barat Province, Sorong (from dealers’ premises); *Cyrtodactylus jellesmae*: MZB lace 4647–4651 Indonesia, Southeast Sulawesi Province, Pulau Buton, Sungai Ladungkula, Kakenauwe Village; *Cyrtodactylus loriae*: SAMA R62635 Papua New Guinea, Kikori Basin, Darai Plateau; SAMA R62636 Papua New Guinea, Eastern Highlands Province, Crater Mountain Wildlife Management Area, Herowana Village; SAMA R62637 Papua New Guinea, Southern Highlands Province, Moro; SAMA R8305, 8369, WAM R67688–676889 Papua New Guinea, Chimbu Province, Karimui Village. *Cyrtodactylus medioclivus*: SAMA R66091 (holotype) Papua New Guinea, Southern Highlands Province, Wanakipa Village; AMS R122441 (paratype), Papua New Guinea, Southern Highlands Province, Bobole; *Cyrtodactylus mimikanus*: BMNH 1946.8.23.5 (syntypes) Indonesia, Papua Province, Mimika River region; MZB lace 3561–3564, Indonesia, Papua Province, Cyclops Mountains, Yongsu; MZB lace 3565–3566 Indonesia, Furu River; MZB lace 2303 (2 specimens) Indonesia, Papua Province, Wapoga River Basin, Siewa. *Cyrtodactylus minor* SAMA R65954 (holotype), SAMA R65953 (paratype) Papua New Guinea, Morobe Province, Huon Peninsula, Tarona Camp, 0.5 km S of Yuwong Village, YUS Tree Kangaroo Conservation Project area; *Cyrtodactylus novaeguineae*: RMNH 2708A–B (cotypes) Indonesia, Papua Province, Triton Bay area; SAMA R66153–4, SJR10338 (field number, currently at SAMA, to be lodged at PNG National Museum) Papua New Guinea, Western Province, upper Strickland basin; SAMA R66156 Papua New Guinea, Western Province, Liddel River; AMS R122410, Papua New Guinea, Southern Highlands Province, Waro; SAMA R62648–9 Papua New Guinea, Gulf Province, Libano; *Cyrtodactylus nuaulu*: MZB lace 2326 (holotype), MZB lace 2325, 2327–2329 (paratypes) Indonesia, Maluku Province, Seram Island, Manusela National Park; *Cyrtodactylus salomonensis*: SAMA R56879 (holotype), SAMA R56780 (paratype) Solomon Islands, Isabel Province, Santa Isabel Island, Kolopakisa; *Cyrtodactylus serratus*: SAMA R62635 Papua New Guinea, Gulf Province, Darai Plateau; SAMA R66155 Papua New Guinea,Western Province, upper Strickland basin; *Cyrtodactylus sermowaiensis*: SAMA R62653 Papua New Guinea, Madang Province, Ramu River basin; *Cyrtodactylus tuberculatus*: SAMA R12058, SAMA 14002 Australia, Queensland, Cooktown; *Cyrtodactylus tripartitus*: SAMA R62638–62644 Papua New Guinea, Milne Bay Province, Misima Island; *Cyrtodactylus zugi*: MZB lace 5574 (holotype), MZB lace 5573, 5575 (paratypes) Indonesia, Papua Barat Province, south coast of Batanta Island; MZB lace 7310 Indonesia, Papua Barat Province, Batanta.
